# Surface-Enhanced Raman Spectroscopic Characterization of Chitosan Extracted from Black Soldier Fly Pupa Shells

**DOI:** 10.1021/acsomega.6c08530

**Published:** 2026-10-26

**Authors:** Nora Slekiene, Zineb Chiki, Ingrida Bruzaite, Juozas Padgurskas, Valentinas Snitka

**Affiliations:** † Pharmacy and Pharmacology Centre, Institute of Biomedical Sciences, Faculty of Medicine, 54694 Vilnius University, M.K. Čiurlionio str. 21/27, Vilnius LT-03101, Lithuania; ‡ Signals, Systems and Components Laboratory (SSCL), Faculty of Sciences and Techniques, 107907 Sidi Mohamed Ben Abdellah University, Route Imouzzer, BP 2202, Fez 30000, Morocco; § Department of Chemistry and Bioengineering, Faculty of Fundamental Sciences, 112678 Vilnius Gediminas Technical University, Sauletekio Av. 11, Vilnius LT-10223, Lithuania; ∥ Faculty of Engineering, Vytautas Magnus University (VMU), Studentų str. 15, Akademija, Kaunas LT-53362, Lithuania; ⊥ Research Centre for Microsystems and Nanotechnology, Kaunas University of Technology, Studentu str. 65, Kaunas LT-51369, Lithuania; # Translational Health Research Institute, Faculty of Medicine, Vilnius University, Žaliųjų Ežerų str. 2, Vilnius LT-08018, Lithuania

## Abstract

Chitosan is widely used as a matrix in metal nanoparticle-based surface-enhanced Raman scattering (SERS) substrates; however, its intrinsic spectral features may interfere with analyte identification. Conventional Raman spectroscopy of chitosan is further limited by strong fluorescence, resulting in scarce, reliable spectral data. In this study, chitosan extracted from Black Soldier Fly (BSF) (*Hermetia illucens*) pupa shells was characterized using X-ray diffraction (XRD), Fourier transform infrared spectroscopy (FTIR), Raman spectroscopy, and silver nanoparticle-enhanced SERS. SERS measurements enabled the acquisition of well-resolved vibrational spectra of chitosan despite the strong fluorescence observed in conventional Raman measurements. Comparison with commercial chitosan and literature data allowed identification of characteristic spectral features associated with the chitosan molecular structure. The synthesized chitosan exhibited distinct structural and spectroscopic characteristics compared with the commercial material, including differences in relative crystallinity and characteristic FTIR and SERS spectral features. The results demonstrate that SERS is a suitable complementary spectroscopic approach for the characterization of chitosan and provides spectral information for the interpretation of chitosan-associated signals in SERS measurements.

## Graphical abstract

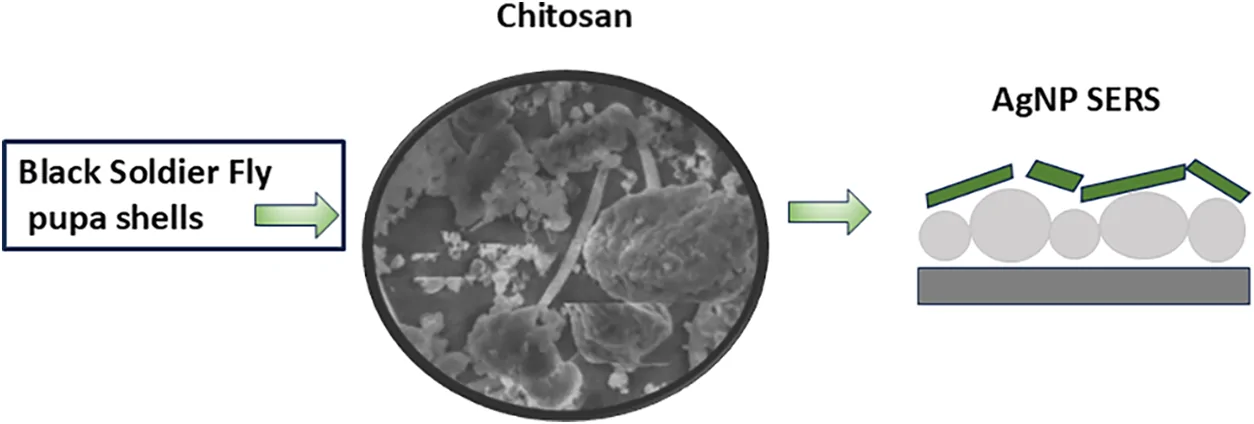

## Introduction

Chitosan, a partially deacetylated derivative of chitin, is one of the most abundant natural polysaccharides after cellulose and has attracted considerable attention because of its biocompatibility, biodegradability, low toxicity, and versatile functional properties. Owing to the presence of reactive amino and hydroxyl groups, chitosan exhibits antimicrobial activity, film-forming ability, and strong affinity for metal ions, which make it useful in a wide range of applications, including drug delivery, tissue engineering, wound healing, environmental remediation, and sensing technologies.^1–3^ Traditionally, chitosan is produced from marine crustacean shells; however, sustainability concerns and an increasing demand have stimulated the search for alternative sources. Insect biomass, particularly Black Soldier Fly (BSF) (*Hermetia illucens*) pupa shells, has recently emerged as a promising renewable resource within circular bioeconomy frameworks, offering comparable physicochemical properties while utilizing industrial insect rearing waste streams.^4^

Current research increasingly focuses on the development of chitosan-based hybrid materials, especially composites incorporating noble metal nanoparticles such as silver and gold. These materials are widely investigated for applications in catalysis, antimicrobial coatings, biosensors, and environmental monitoring.^5–7^ Chitosan–metal nanoparticle assemblies have attracted significant interest as substrates for surface-enhanced Raman scattering (SERS) spectroscopy. SERS enables highly sensitive molecular detection by amplifying Raman signals through localized surface plasmon resonances of metallic nanostructures, often achieving enhancement factors of several orders of magnitude.^8–10^ Chitosan is well-suited as a supporting matrix because its functional groups can coordinate metal ions, stabilize nanoparticles, and promote the formation of porous three-dimensional networks rich in electromagnetic “hot spots”.^11–13^ For example, chitosan-based nanocomposites incorporating silver or gold nanoparticles have demonstrated improved signal intensity, reproducibility, and stability in SERS sensing applications.^5,6,14–16^

Despite these advantages, chitosan-based SERS substrates present important analytical challenges. The recorded spectrum often contains contributions from both the analyte and chitosan matrix itself. Because chitosan possesses intrinsic vibrational features, its spectral peaks may overlap with those of the target molecules, complicating interpretation and potentially leading to ambiguous results. Reliable identification of the intrinsic spectral signature of chitosan is therefore essential for an accurate analysis of hybrid chitosan-based sensors.

Conventional Raman spectroscopy, however, provides limited information about chitosan due to a strong fluorescence background induced by laser excitation, which frequently obscures the weak Raman signal. Consequently, high-quality Raman spectra of chitosan have been scarce in the literature. Surface-enhanced Raman scattering offers a promising alternative, as plasmonic nanostructures not only enhance Raman scattering but also suppress fluorescence, enabling acquisition of informative spectra that reflect molecular structure.^8^ Nevertheless, although chitosan-based SERS substrates have been extensively studied for sensing applications, the use of SERS specifically for the structural characterization of chitosan itself and its derivatives remains largely unexplored.

Furthermore, the physicochemical properties of chitosan strongly depend on its source and extraction conditions, which influence parameters such as the molecular weight, degree of deacetylation (DD), crystallinity, and impurity content. Insect-derived chitosan, particularly from black soldier fly pupa shells, represents a sustainable alternative to marine sources, but its structural characteristics and spectroscopic fingerprints are not yet fully understood. Although the extraction and conventional physicochemical characterization of chitin and chitosan from *H. illucens* have been previously reported, detailed SERS characterization of the intrinsic vibrational features of insect-derived chitosan remains limited. A comparative analysis combining X-ray diffraction (XRD), Fourier transform infrared spectroscopy (FTIR), conventional Raman spectroscopy, and SERS may therefore provide complementary information about the structural and spectroscopic characteristics of this material.

The problem addressed in this study is the limited availability of reliable vibrational spectral information for chitosan, particularly insect-derived chitosan, because strong fluorescence can obscure conventional Raman spectra, while the intrinsic spectral contribution of chitosan itself may complicate the interpretation of chitosan-containing SERS systems.

Therefore, the present study aims to characterize chitosan synthesized from black soldier fly pupa shells using complementary XRD, FTIR, Raman, and SERS techniques and to compare its structural and spectroscopic characteristics with those of commercial chitosan. Emphasis is placed on obtaining and interpreting the intrinsic SERS spectral signature of chitosan, which is important because chitosan itself may contribute to spectral features in chitosan-containing SERS systems. This approach provides a direct comparison between insect-derived and commercial chitosan and establishes spectral reference information for the interpretation of chitosan-associated SERS signals.

## Materials and Methods

### Rearing of *H. illucens*

*H. illucens* eggs, purchased from an ECHO Asia Small Farm Resource Centre in Chiang Mai, Thailand, were hatched under controlled laboratory conditions. The larvae were reared in shallow trays containing wheat bran as the organic substrate, maintained at 28 °C and 60–70% relative humidity. Upon reaching the prepupal stage, the larvae were allowed to migrate to a dry area for pupation. The pupa was then transferred to a mesh cage with a photoperiod of 14–16 h of light per day to facilitate adult emergence and mating. Females were provided with corrugated cardboard as oviposition material near the food source. Regular misting ensured adequate hydration for adults, and all containers were cleaned regularly to maintain hygiene and colony health.

### Sample Collection and Pretreatment

Black Soldier Fly (BSF) pupa shells were collected from a culture described in the paragraph above at Vytautas Magnus University, Lithuania, in October 2024. The pupa shells were dried in the oven for 48 h at 60 °C to eliminate water. The pupa samples were transformed directly into the chitin treatment step without grinding (we kept the pupa’s original shape).

The main chemicals used to extract chitin and chitosan during this study were hydrochloric acid (HCl, 37%) and sodium hydroxide (pellets, 97%).

### Chitosan Production from BSF

The chitosan production process consists of three stages: demineralization, deproteinization, and deacetylation. BSF was demineralized by adding a 2 M HCl solution for 90 min at 120 °C in a sealed autoclave under autogenous pressure. The residue was washed with distilled water until a neutral pH (approximately 7) was reached and then dried in an oven at 60 °C for 24 h. The demineralization residue is deproteinized with an alkaline solution (3 M NaOH) at a ratio of 1:10 (w/v) and then neutralized with distilled water until pH = 7. The chitin yield, calculated relative to the initial dry mass of BSF biomass, was 22.9%. The last step in this production process is deacetylation, which consists of adding a NaOH solution (60%) to the purified chitin at a temperature of 100 °C for 2 h. Finally, the resulting chitosan was frozen below −40 °C and subsequently lyophilized under vacuum for 48 h to obtain a dry material suitable for subsequent characterization (Figure 1). The chitosan yield, calculated relative to the mass of purified chitin used for deacetylation, was 80.87%. This process ensured the complete removal of moisture while preserving the structural integrity and quality of the chitosan.

**Figure 1:**
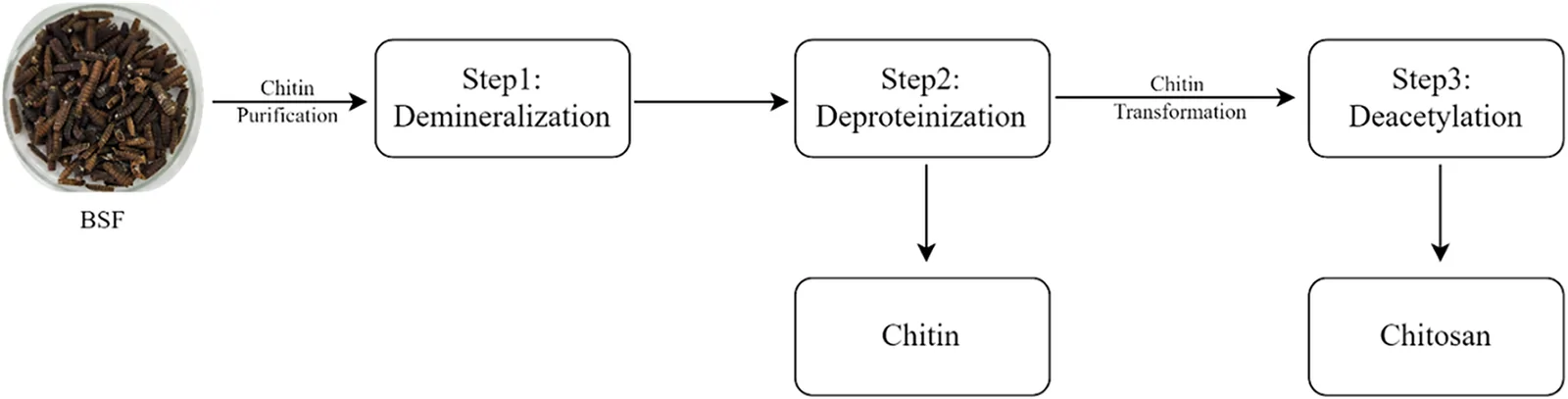
Chitosan production process.

The medium-molecular-weight chitosan powder (density 1 g/cm^3^, PCode 102803879) used for reference spectra investigation was purchased from Sigma-Aldrich.

### Attenuated Total Reflectance Infrared Absorption Spectroscopy (ATR-FTIR)

Infrared spectrum was collected using an infrared absorption spectrometer α (Bruker, Inc., Germany) equipped with a room-temperature DLATGS detector and ATR accessory (Platinum ATR Diamond). Spectra were collected with 4 cm^–1^ resolution from 100 interferogram scans.

### Determination of the Degree of Deacetylation (DD)

The degree of deacetylation (DD) of the synthesized chitosan was estimated from baseline-corrected FTIR spectra using established absorbance-ratio methods. Two approaches were evaluated. The first approach was based on the method described by Baxter et al.^17^ using the absorbance ratio of the amide I band at 1655 cm^–1^ to the O–H stretching band at 3450 cm^–1^. The degree of acetylation (DA) and the corresponding DD were calculated as

$$\mathrm{DA}\;\left(\%\right)=\left(A_{1655}/A_{3450}\right)\times 115$$

DD(%)=100−DA(%)

The second approach was based on the method described by Brugnerotto et al.,^18^ using the absorbance ratio of the amide III band at 1320 cm^–1^ to the reference band at 1420 cm^–1^. DA and DD were calculated as

$$\mathrm{DA}\;\left(\%\right)=31.92\times \left(A_{1320}/A_{1420}\right)-12.20$$

DD(%)=100−DA(%)

For both methods, absorbance values at the corresponding wavenumbers were obtained from the baseline-corrected FTIR spectrum. Both approaches were evaluated because FTIR-based estimates of DD may depend on the selected absorption and reference bands and can be influenced by spectral overlap and baseline variations, particularly in the broad O–H/N–H stretching region.

### X-ray Diffraction (XRD)

Powder X-ray diffraction (XRD) patterns were collected using a Rigaku SmartLab diffractometer equipped with a Ge(400) two-bounce monochromator and an SC-70 scintillation detector. Measurements were performed using Cu Kα radiation (λ = 1.5406 Å) in Bragg–Brentano geometry. The diffraction patterns were recorded over a 2θ range of 5–75°, using a step size of 0.02° and a scan rate of 0.2–0.5° min^–1^.

The relative crystallinity of the samples was estimated from the XRD patterns using a peak-height crystallinity index approach.^19^ The crystallinity index (CrI) was calculated according to

$$\mathrm{CrI}\;\left(\%\right)=\left[\left(I_{\mathrm{cr}}-I_{am}\right)/I_{\mathrm{cr}}\right]\times 100$$

where *I*_cr_ is the maximum intensity of the principal crystalline diffraction peak and *I*_am_ is the intensity of the amorphous contribution. The same procedure was applied consistently to the synthesized and commercial chitosan samples.

### Scanning Electron Microscopy (SEM)

Detection and characterization of the size, shape, and surface of synthesized and commercially available chitosan samples were performed by using a Thermo Fisher Scientific Quattro S4 high-resolution scanning electron microscope. All measurements were conducted in a high-vacuum mode. Elemental analysis was conducted using an energy-dispersive X-ray spectroscopy (EDS) system, UltraDry 129 eV 30.

### Surface-Enhanced Raman Scattering (SERS) Spectroscopy

The synthesized chitosan exhibited a broad particle size distribution ranging from hundreds of micrometers to the nanometer scale. Before Raman and SERS measurements, 5 mg of synthesized chitosan was dispersed in 20 mL of distilled water. The dispersion was sonicated in an ultrasonic bath for 5 min at room temperature and subsequently allowed to settle for 10 min. After sedimentation of the larger particles, a suspension enriched in smaller particles was collected by pipetting from the middle region of the tube, as illustrated in Figure S1 (Supporting Information). No centrifugation or chemical precipitation step was used. One pipetted drop (∼20 to 50 μL) of the resulting suspension was deposited onto the AgNP SERS substrate and allowed to dry completely before spectral acquisition.

Raman and SERS measurements were performed using an NT-MDT NTEGRA Spectra system in an inverted configuration, equipped with a 100× objective (NA = 0.7) and a thermoelectrically cooled CCD detector (−60 °C). Conventional Raman spectra were recorded using an excitation wavelength of 532 nm, a laser power of 30 mW, and an acquisition time of 90 s. SERS spectra were recorded using 532 nm excitation at a laser power of 0.1 mW at the sample surface and an acquisition time of 5 s. All spectra were calibrated against the first-order Si phonon band at 520 cm^–1^. The spectral resolution was 1.1 cm^–1^.

To assess spatial reproducibility, 25 spectra were acquired at different positions on the SERS substrate by moving the sample relative to the excitation laser beam using the X–Y positioning stage. These measurements, therefore, represent spatially resolved measurements from different positions of the same prepared sample rather than independent sample preparations.

Spectral processing and analysis were performed using Spectragryph version 1.2.16 (Spectragryph—Optical Spectroscopy Software) and Origin 8.5 (OriginLab Corporation, Northampton, MA, USA).

### Preparation of SERS Substrates

Silver nanoparticle SERS substrates were used in this work. Silver nanoparticles (AgNPs) deposition on n-type (111) silicon wafer chips substrate was fabricated through direct reduction of silver ions by silicon, as reported by our research group,^20^ and briefly described here. Silicon wafers were sliced into 1.5 cm × 1.5 cm pieces, cleaned by immersion in pure ethanol, and dried with a nitrogen stream. Hydrofluoric acid (HF) was diluted in distilled water to achieve a 24% HF concentration. The silver precursor solution consisted of AgNO_3_ diluted in water to 10.0 mM. The prepared HF solution and AgNO_3_ solution were mixed 1:1 v/v. The silicon wafers were immersed in the reaction mixture for 10 s, then immediately transferred to the pure water and finally dried under a nitrogen flow. The formation mechanism of silver nanoparticles can be described by the following equations

$$2{\mathrm{Ag}}^{+}+2e^{-}\to 2{\mathrm{Ag}}^{0}\left(s\right)$$

$$2H^{+}+2e^{-}\to H_{2}$$

$$\mathrm{Si}+6F^{-}\to {\mathrm{SiF}}_{6}^{2-}+4e^{-}$$

$$\mathrm{Si}+2{\mathrm{Ag}}^{+}+6F^{-}+2H^{+}\to {\mathrm{SiF}}_{6}^{2-}+2\mathrm{Ag}+H_{2}$$

The prepared SERS substrates were used immediately after fabrication to minimize possible changes in the AgNP surface. Detailed characterization of AgNP substrates prepared using this protocol, including atomic force microscopy (AFM), scanning electron microscopy (SEM), and UV–vis spectroscopy, was reported in our previous study.^21^

### AFM Characterization of SERS Substrates

The morphology of the prepared SERS substrates was characterized by atomic force microscopy (AFM). Ten independently prepared SERS substrates were analyzed to assess substrate-to-substrate morphological reproducibility. AgNP height distributions were obtained from the AFM images, and the mean nanoparticle height and corresponding standard deviation (SD) were calculated from the measurements. The standard deviation was calculated according to the following equation

$$SD=\sqrt{\frac{\sum {\left(x-\mathrm{x̅}\right)}^{2}}{N-1}}$$

where SD is the standard deviation, *N* is the number of values in the data set, *x* is each value of the histogram, and *x̅* is the mean value of the histograms of 10 samples.

## Results and Discussion

### Morphological and Elemental Characterization of Commercial and Synthesized Chitosan

The morphology and elemental composition of commercial medium-molecular-weight chitosan and synthesized chitosan were investigated using scanning electron microscopy (SEM) and energy-dispersive X-ray spectroscopy (EDX). Representative SEM images and EDX spectra are shown in Figures 2 and 3.

**Figure 2:**
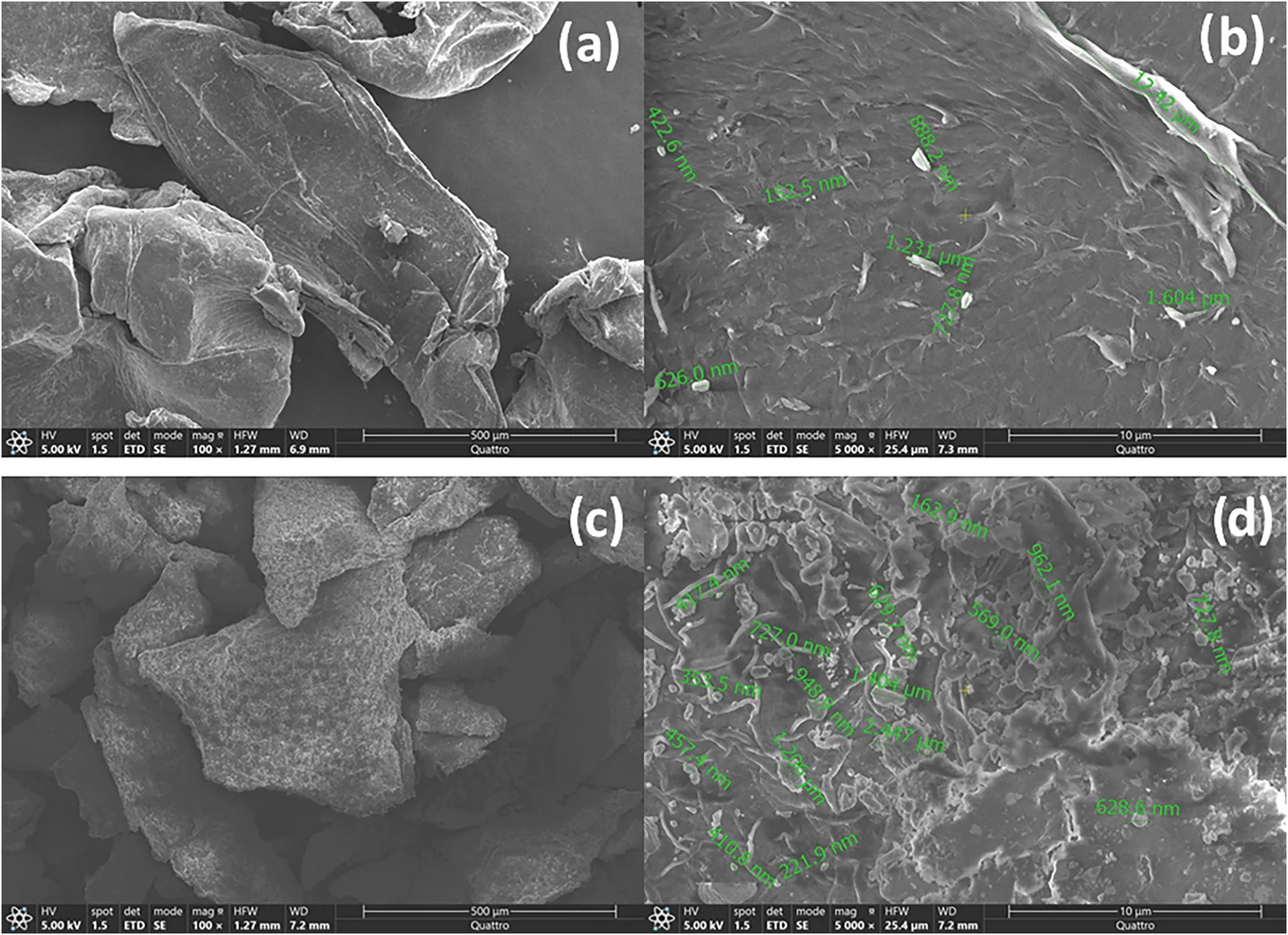
SEM image of commercial chitosan macroflakes (a) and nanoparticles (b), and from Black Soldier Fly pupa shells macroflakes (c) and nanoparticles (d).

**Figure 3:**
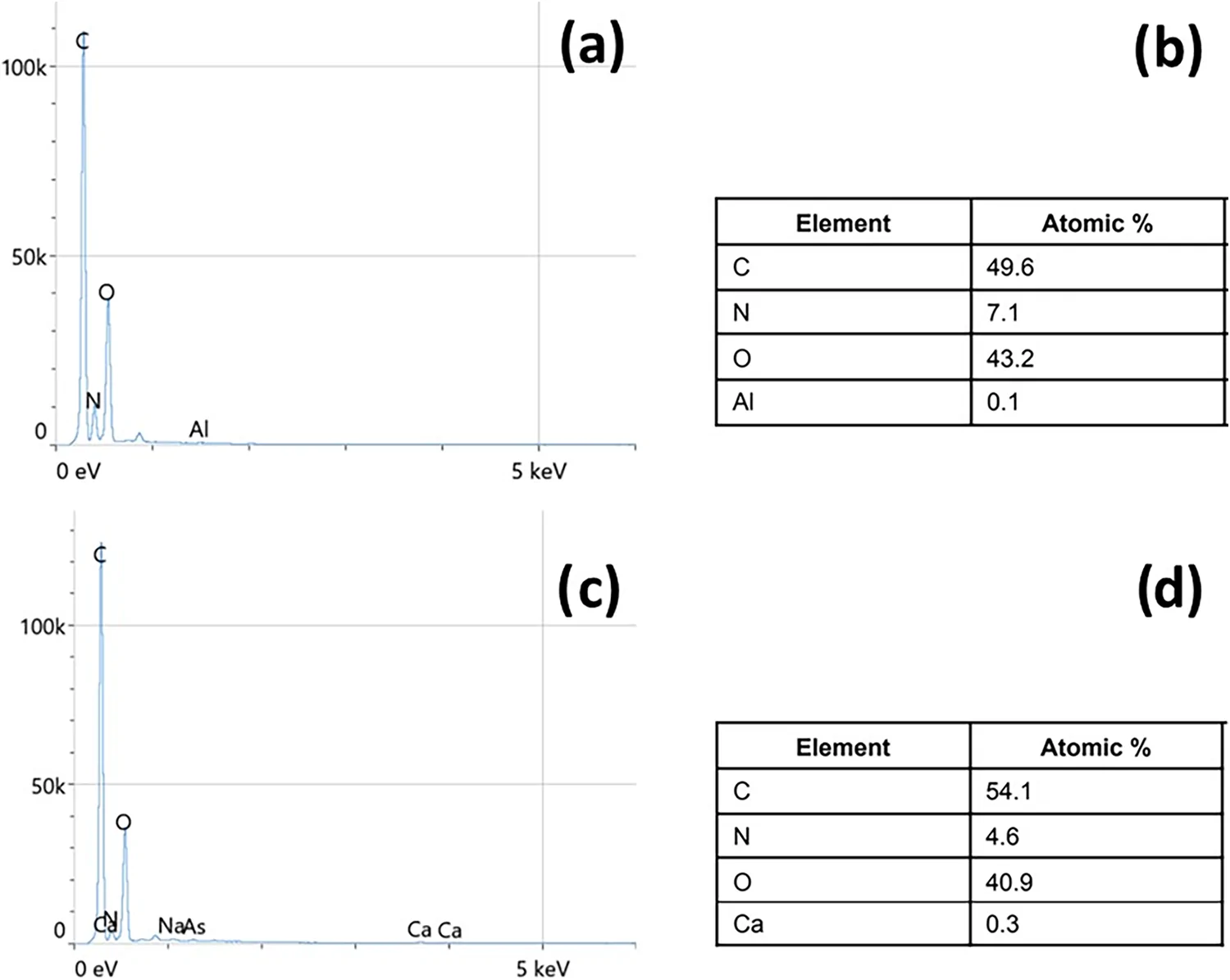
EDX of commercial chitosan atomic spectra (a) and composition (b), and from Black Soldier Fly pupa shells atomic spectra (c) and composition (d).

Commercial chitosan predominantly consisted of large compact macroflake-like structures with only a small number of rarely observed flakes ranging from approximately 400 nm to 1.5 μm (Figure 2a,b). In contrast, the synthesized chitosan exhibited a more porous and fragmented morphology composed of smaller macroflakes and abundant nanoflake-like structures, with dominant particle sizes ranging from approximately 100 to 600 nm (Figure 2c,d). Larger aggregates reaching several micrometers were also observed in both samples.

EDX analysis confirmed that both samples were primarily composed of carbon, nitrogen, and oxygen, which is characteristic of chitosan materials due to the presence of glucosamine and *N*-acetylglucosamine units in the polymer backbone (Figure 3). Minor elemental impurities detected in both samples are likely associated with residual inorganic compounds or sample preparation artifacts. The differences in elemental composition between the commercial and synthesized samples may reflect differences in the sample origin, processing conditions, and presence of residual inorganic components. These EDX data are interpreted as comparative elemental characterization and are not used independently to determine the degree of deacetylation or the specific chemical states. Previous studies have similarly shown that the extraction and processing conditions of chitosan can influence its surface morphology and elemental composition.^4,22^

Overall, SEM and EDX analyses demonstrate clear differences between the commercial and the synthesized chitosan samples. The commercial material was characterized by larger and more compact macroflakes with only rare nanoscale fragments, whereas the synthesized chitosan exhibited a more porous morphology and a higher abundance of nanoflake-like structures. These observations demonstrate clear morphological differences between the commercial and synthesized chitosan samples, particularly in terms of particle size, surface morphology, and abundance of smaller flake-like structures.

### Structural Characterization of Commercial and Synthesized Chitosan by XRD and FTIR

The XRD patterns of both commercial and synthesized chitosan samples exhibited characteristic reflections of semicrystalline chitosan structures (Figure 4a). The synthesized chitosan showed major diffraction peaks at 2θ = 9.40 and 19.36°, whereas the corresponding peaks of the commercial chitosan were observed at 2θ = 9.86 and 19.96°, respectively. These diffraction features are consistent with the presence of ordered and less ordered regions within the chitosan structure.^23^

**Figure 4:**
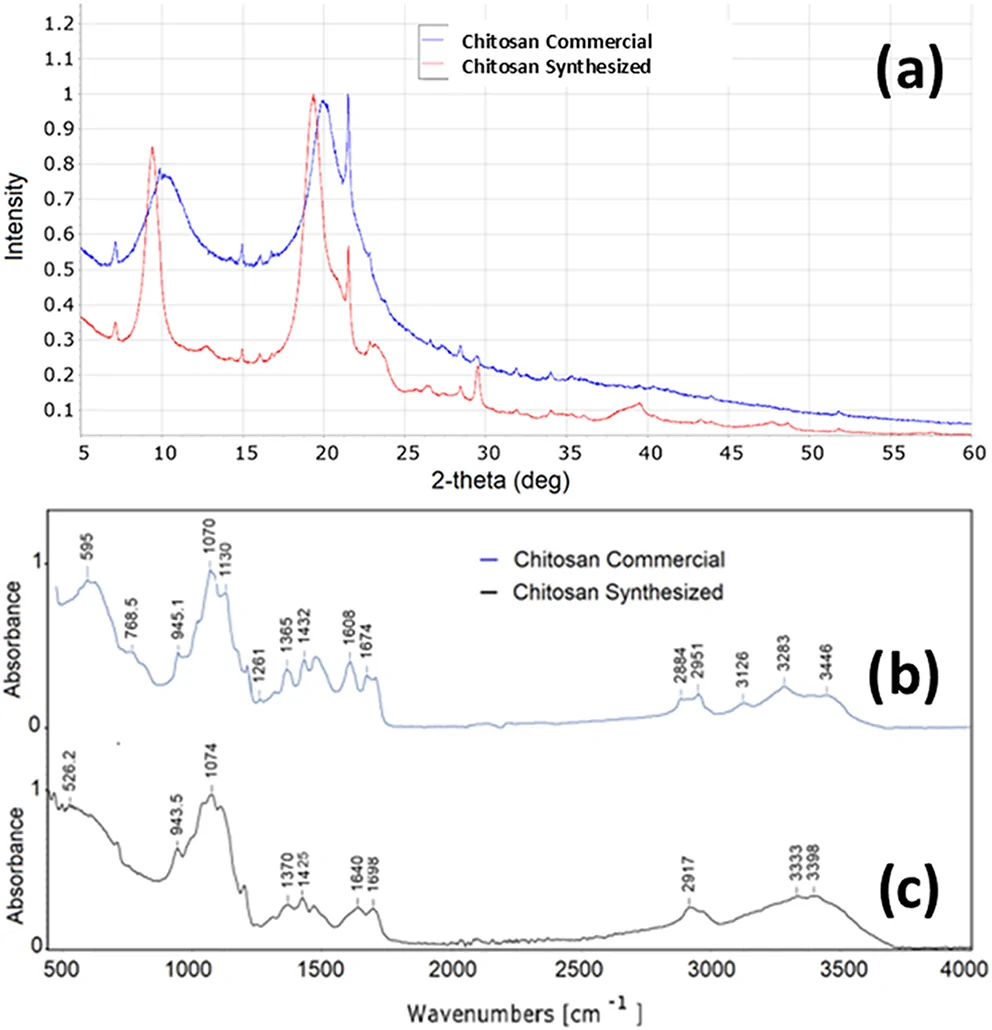
XRD patterns of commercial and synthesized chitosan (a), and FTIR spectra of commercial chitosan (b) and chitosan synthesized from Black Soldier Fly pupa shells (c). The FTIR spectra were recorded in the range of 4000–1300 cm^–1^.

The relative crystallinity of the two samples was quantitatively compared using the peak-height crystallinity index (CrI) approach described in the Materials and Methods section. The principal crystalline diffraction maximum used for the calculation was observed at 2θ = 19.36° for the synthesized chitosan and 19.96° for the commercial chitosan, while the amorphous contribution was evaluated at 2θ = 15.32° for both samples (Table 1).

**Table 1:** XRD Parameters Used for the Determination of the Crystallinity Index of Synthesized and Commercial Chitosan

sample	principal crystalline peak, 2θ (°)	*I*_cr_ (counts)	amorphous contribution, 2θ (°)	*I*_am_ (counts)	CrI (%)
synthesized chitosan	19.36	74,028	15.32	17,315	76.61
commercial chitosan	19.96	46,206	15.32	26,643	42.3

The calculated CrI values were 76.61% for the synthesized chitosan and 42.3% for the commercial chitosan. Thus, according to this XRD-derived relative crystallinity parameter, the synthesized chitosan exhibited a higher degree of structural ordering than that of the commercial sample. The differences in the positions and relative intensities of the principal diffraction features indicate differences in the organization of the chitosan chains and in the relative contributions of ordered and less-ordered regions within the two materials. In particular, the substantially higher CrI of the synthesized chitosan indicates a greater relative contribution of ordered domains under the applied XRD evaluation procedures. These structural differences may be related to differences in the sample origin and processing history, including the extraction and deacetylation conditions used for the synthesized material. However, because the peak height CrI represents a relative XRD-derived parameter, the calculated values should not be interpreted as absolute crystalline-phase fractions.

XRD analysis revealed a clear difference in the relative crystallinity of the two chitosan samples. On the basis of the consistently applied peak-height CrI approach (Figure 4a), the synthesized chitosan exhibited a higher relative crystallinity (76.61%) than the commercial chitosan (42.3%). This difference indicates a greater contribution of the ordered regions to the XRD profile of the synthesized sample. Nevertheless, the CrI values represent relative XRD-derived parameters and should not be interpreted as absolute crystalline-phase fractions.

### Fourier-Transform Infrared (FTIR) Spectroscopy Analysis

Fourier-transform infrared (FTIR) spectroscopy was performed to confirm the chemical identity, evaluate the efficacy of the *N*-deacetylation reaction, and assess the structural integrity, protonation state, and crystalline order of the synthesized chitosan in comparison to commercial chitosan standards. Peak assignments highlight functional group conversions, degree of deacetylation (DD%), and protonation state characteristic of the deacetylated polysaccharide backbone.

FTIR spectra of both commercial and synthesized chitosan samples demonstrated characteristic absorption bands associated with chitosan functional groups and polysaccharide structure (Figure 4b,c).

Successful *N*-deacetylation of chitin into chitosan was confirmed by examining the amide spectral region. Residual *N*-acetylated units were identified by the weak Amide I absorption band at 1655 cm^–1^ (C=O carbonyl stretching). Crucially, the spectrum exhibited a prominent Amide II band around 1580–1600 cm^–1^, attributed to the in-plane N–H deformation bending of free primary amine (−NH_2_) groups. The elevated intensity of the 1580 cm^–1^ band relative to the 1655 cm^–1^ band serves as spectroscopic verification of the cleavage of *N*-acetyl linkages.

Lower wavenumber vibrations confirmed the preservation of the carbohydrate ring system. Bending vibrations of C–H and C–N modes appeared in the region of 1420–1380 cm^–1^. Additionally, intense envelope absorptions spanning 1080–1030 cm^–1^ were assigned to skeletal C–O–C asymmetric stretching of the β-glycosidic linkage and endocyclic C–O vibrations.

### Data Analysis and Functional Group Confirmation in Commercial and Synthesized Chitosan Samples

The FTIR spectra of both commercial and synthesized chitosan exhibited the characteristic absorption bands associated with the main functional groups of the chitosan structure. The broad absorption band in the 3400–3300 cm^–1^ region is attributed to overlapping O–H and N–H stretching vibrations and reflects the hydrogen-bonding interactions within the polymer structure. The band observed at approximately 1580–1600 cm^–1^ is mainly associated with N–H bending vibrations of primary amino groups and is consistent with the deacetylated structure of chitosan. At the same time, the absorption band near 1650 cm^–1^, assigned predominantly to the C=O stretching vibration of amide I, indicates the presence of residual *N*-acetylglucosamine units. Differences in the position and relative intensity of these amide- and amino-associated bands between the commercial and synthesized samples may reflect differences in residual acetyl-group content and local molecular environments. The strong absorption bands in the 1080–1030 cm^–1^ region are associated with C–O and C–O–C stretching vibrations, including contributions from glycosidic linkages and saccharide-ring vibrations, and are consistent with preservation of the polysaccharide backbone. Overall, the FTIR spectra are consistent with the characteristic functional-group pattern of chitosan and reveal differences in the local molecular environments of the commercial and synthesized materials. Similar FTIR features have been widely reported for semicrystalline chitosan materials.^18,24^ The characteristic band positions, relative normalized intensities, and corresponding vibrational assignments are summarized in Table 2.

**Table 2:** FTIR Band Positions (cm^–1^), Assignments, and Relative Normalized Intensities for Synthesized and Commercial Chitosan

band (functional group)	synthesized chitosan (cm^–1^)	relative intensity^a^	commercial chitosan (cm^–1^)	relative intensity^a^	assignment
O–H/N–H stretching	∼3400 to 3445	0.355	∼3283	0.335	hydrogen-bonded O–H and N–H stretching vibrations
C–H stretching	∼2920	0.283	∼2951	0.295	C–H stretching vibrations of the aliphatic ring/CH_2_ groups
amide I (C=O)	∼1640	0.283	∼1608	0.479	C=O stretching of acetylated groups indicative of residual *N*-acetyl groups
amide II/N–H (amines)	∼1600	0.235	∼1600	0.456	N–H bending and C–N stretching; free −NH_2_ contributions
C–H bending	∼1425	0.345	∼1432	0.488	CH_2_/CH_3_ bending vibrations
amide III	∼1350	0.281	∼1350	0.364	C–N stretching coupled with N–H bending
C–O/C–O–C stretching (1)	∼1075	1.000	∼1070	1.000	C–O and C–O–C stretching in the pyranose ring
C–O/C–O–C stretching (2)	∼1041	0.948	∼1050	0.858	polysaccharide backbone C–O stretching

a Intensities are normalized to the maximum absorbance in the 1800–900 cm^–1^ region for each spectrum.

Despite the overall similarity of the FTIR spectra, several differences between the commercial and synthesized chitosan samples were observed. The synthesized chitosan exhibited broader and shifted absorption bands, particularly in the O–H/N–H stretching region, with the main band centered at approximately 3398 cm^–1^ compared with approximately 3283 cm^–1^ for the commercial sample. In chitosan, differences in the position and width of this broad band may reflect variations in the strength and distribution of hydrogen-bonding interactions between the two materials.

Differences were also observed in the amide I and amide II/amine regions (≈1700–1500 cm^–1^), where the synthesized material showed broader and less resolved overlapping features, indicating differences in the local environments of C=O– and N–H-containing groups. In the fingerprint region (≈1150–950 cm^–1^), the synthesized chitosan exhibited fewer well-resolved C–O/C–O–C bands than the commercial sample. These differences in band positions, widths, and multiplicity may reflect variations in hydrogen-bonding interactions, pyranose-ring conformation, interchain interactions, and local molecular organization between the two chitosan samples.

Taken together, these spectral differences indicate variations in intermolecular interactions, residual acetyl-group environment, structural heterogeneity, and hydrogen-bond organization between the two samples. Specifically, the broader and shifted bands of the synthesized chitosan may reflect a less-ordered hydrogen-bond network and higher local structural heterogeneity compared with the commercial chitosan.

### Degree of Deacetylation

The degree of deacetylation of the synthesized chitosan was estimated using the two FTIR absorbance-ratio approaches described in the Materials and Methods section. The Baxter method, based on the *A*_1655_/*A*_3450_ ratio, yielded a value outside the physically meaningful range and was therefore not used for the final DD estimation. This result may reflect the sensitivity of the broad O–H/N–H stretching region to baseline selection and moisture-related contributions.

The Brugnerotto method, based on the *A*_1320_/*A*_1420_ ratio, was therefore used for the final FTIR-based estimation. The baseline-corrected absorbance values were *A*_1320_ = 0.21 and *A*_1420_ = 0.33, corresponding to an *A*_1320_/*A*_1420_ ratio of 0.636. Using the Brugnerotto relationship, the degree of acetylation (DA) was estimated to be approximately 8.1%, corresponding to a degree of deacetylation (DD) of ∼91.9%. This result indicates the extensive deacetylation of the synthesized chitosan.

### Raman and Surface-Enhanced Raman Spectroscopy Analysis of Commercial and Synthesized Chitosan

To complement the infrared absorption data, SERS spectroscopy was conducted utilizing plasmonic silver nanoparticles (AgNPs) on Si as active substrates. SERS addresses the weak inelastic Raman scattering cross sections of biopolymers, providing detailed structural insights into local surface conformations, functional group interactions with metal surfaces, and subtle variations between synthesized and commercial chitosan samples.

The morphology and optical properties of the prepared AgNP-based SERS substrates were characterized by AFM and UV–Vis spectroscopy (Figure 5a–c). AFM analysis revealed the formation of approximately spherical Ag nanoparticles with a relatively narrow height distribution (Figure 5a). The mean AgNP heights determined for 10 independently prepared SERS substrates ranged from 56 to 68 nm, with an overall mean height of approximately 61 nm and a standard deviation of 4 nm (Figure 5b), indicating relatively low substrate-to-substrate variation in nanoparticle height. The UV–Vis absorption spectrum exhibited a broad plasmon resonance band with a maximum at approximately 447 nm (Figure 5c), consistent with the optical response of Ag nanostructures. Together, the AFM and UV–Vis results provide complementary morphological and optical characterization of the AgNP-based substrates used for SERS measurements.

**Figure 5:**
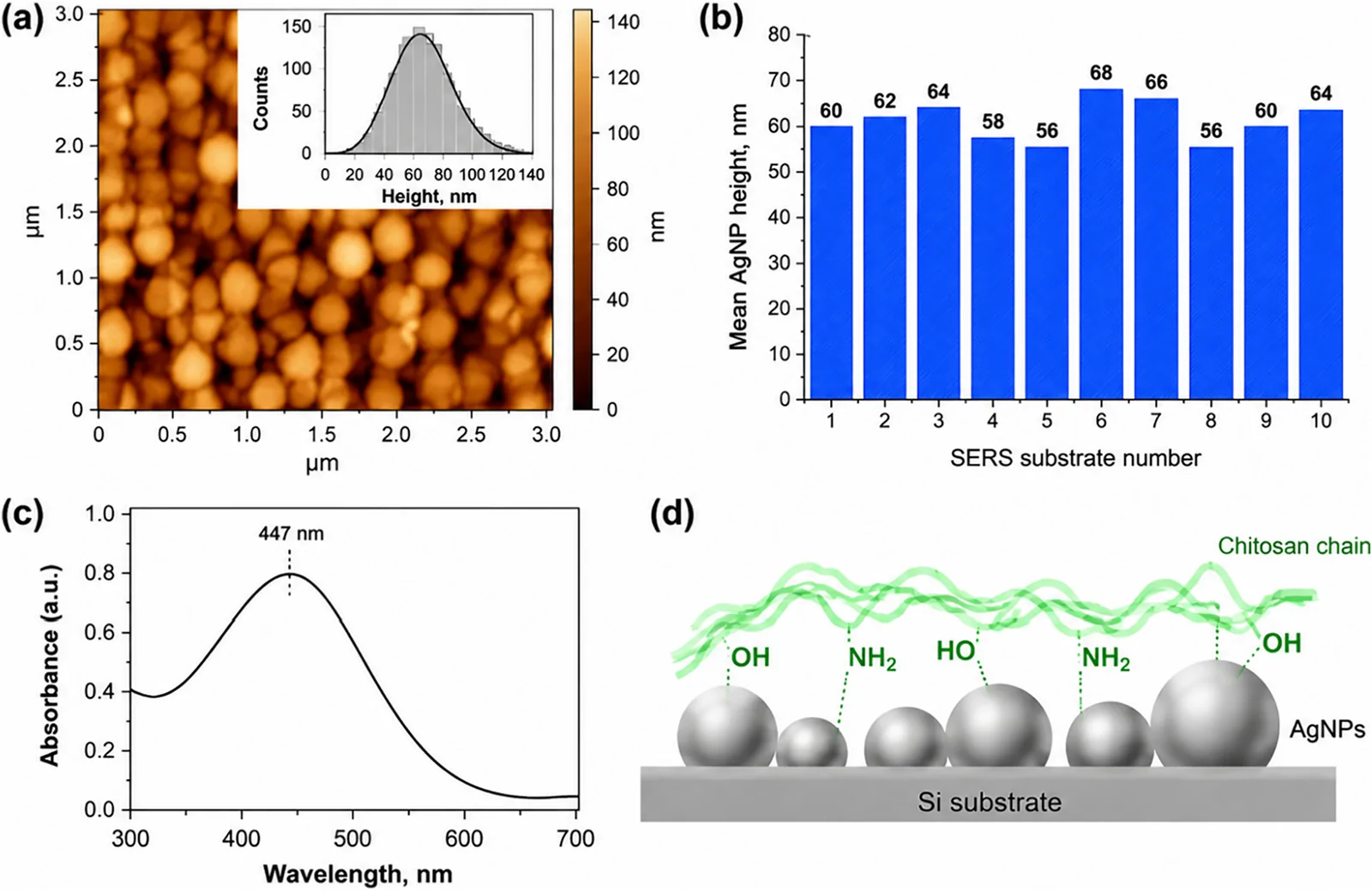
Characterization of the AgNP-based SERS substrates and schematic representation of chitosan interaction with the substrate: (a) representative AFM image of the AgNP surface with the corresponding nanoparticle height-distribution histogram (inset); (b) mean AgNP height determined for each of 10 independently prepared SERS substrates; (c) the UV–Vis absorption spectrum of the AgNP-based SERS substrate showing the localized surface plasmon resonance band centered at approximately 447 nm; and (d) schematic representation of the proposed multipoint interaction of chitosan chains with the AgNP surface.

On the basis of the SERS spectral features, a possible multipoint interaction of chitosan with the AgNP surface is proposed (Figure 5d). The simultaneous enhancement of vibrations associated with amino groups and the polysaccharide backbone suggests that amino groups may act as important anchoring sites, while the flexibility of the chitosan chain may allow adjacent backbone segments to approach the AgNP surface. This model is therefore more consistent with multiple surface contact points than with a uniformly flat orientation of the entire polymer chain. However, the schematic is intended only as a conceptual representation since the present SERS measurements do not allow the exact adsorption geometry or orientation of the chitosan chains to be determined.

To assess the spatial reproducibility of the SERS response across the substrate, we acquired 25 spectra at different positions for each chitosan sample. The mean spectra together with the corresponding standard deviations are presented in Figure 6. These measurements characterize spot-to-spot variability across the SERS-active surface and are not considered to be independent experimental replicates.

**Figure 6:**
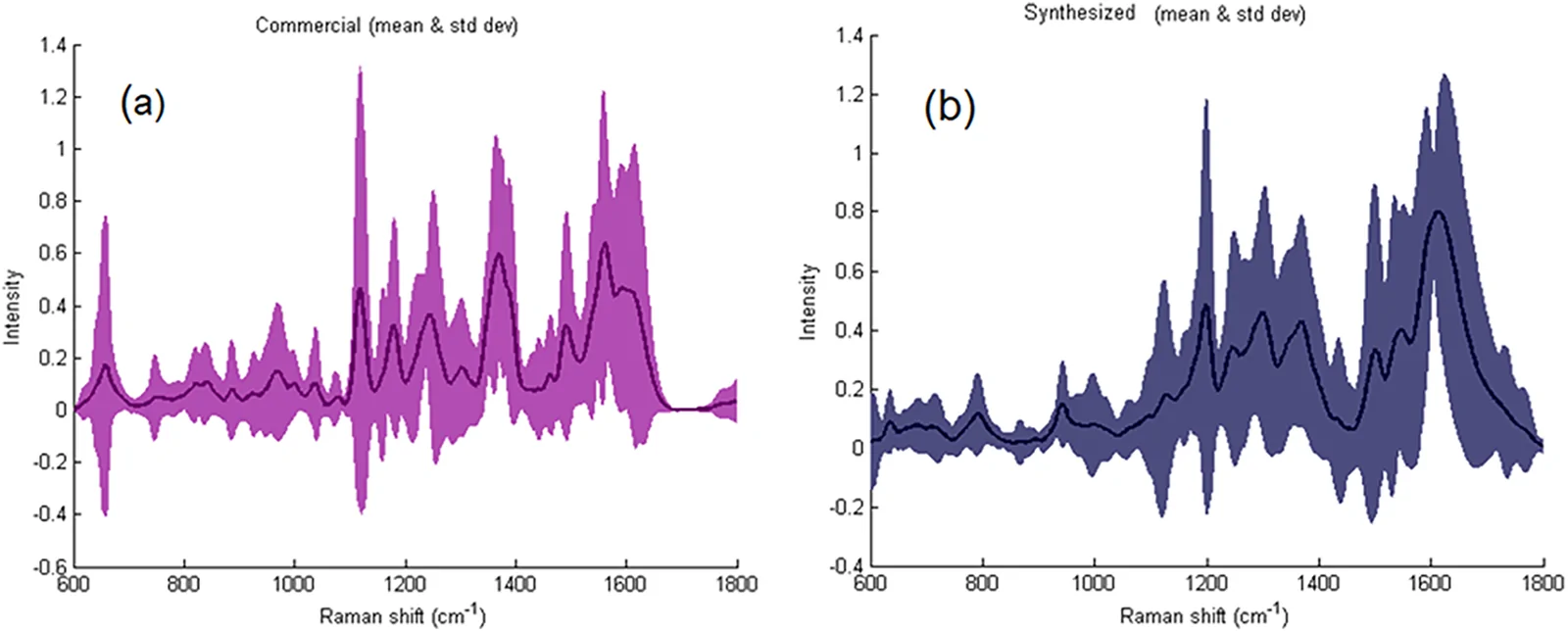
Mean SERS spectra of (a) commercial and (b) synthesized chitosan obtained from measurements at 25 different positions on the SERS substrate. The corresponding standard deviations represent spot-to-spot variability of the SERS response.

The data demonstrate noticeable spot-to-spot variation in the SERS band intensities. Such variability is expected for SERS measurements because the electromagnetic enhancement is highly sensitive to the local nanoscale morphology of the metallic substrate, including variations in the hotspot density, interparticle spacing, and nanoparticle geometry. In addition, differences in the number and orientation of molecules interacting with the SERS-active regions may contribute to the observed intensity variability. Therefore, the mean spectra and their standard deviations obtained from 25 spatially distributed measurements were used to characterize the spatial variability of the SERS response rather than relying on individual representative spectra.^25,26^

In our work, Raman spectroscopy was used to investigate the molecular structure of chitosan samples in the fingerprint region (∼400 to 1600 cm^–1^), where the characteristic vibrational modes of polysaccharides are typically observed (Figure 7a,b). Conventional Raman spectra of chitosan samples exhibited a strong fluorescence background, which significantly reduced spectral clarity and partially masked weaker vibrational bands. Experimentally obtained Raman and SERS spectra are presented in Figure 7.

**Figure 7:**
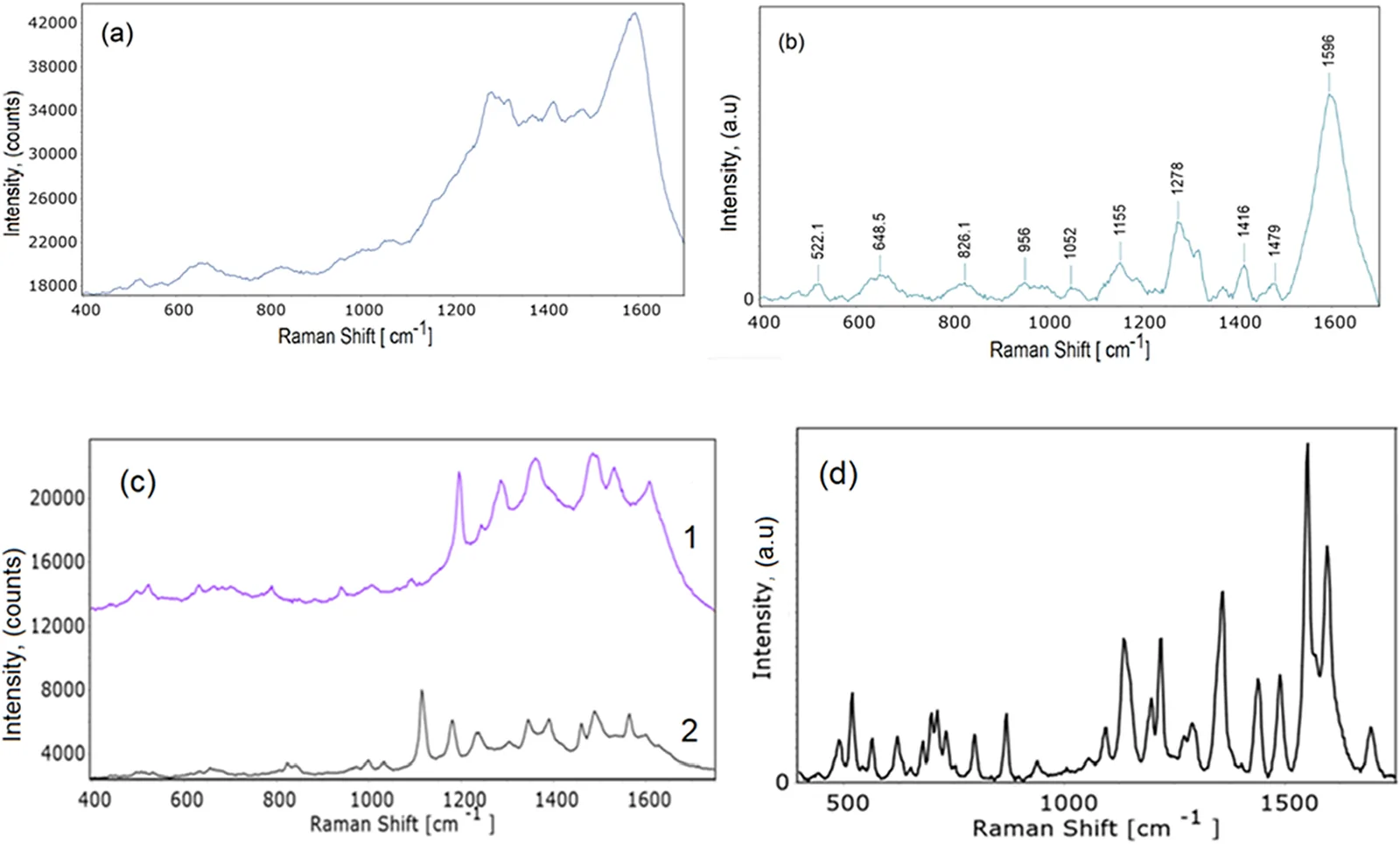
Comparative Raman and SERS spectra of commercial and synthesized chitosan samples recorded in the region 400–1600 cm^–1^: (a) raw Raman spectra of commercial chitosan on Si substrate (excitation wavelength 532 nm); (b) Raman spectra after fluorescence background removal; (c) raw SERS spectra (excitation wavelength 532 nm) of commercial chitosan (2) and synthesized chitosan from Black Soldier Fly pupa shells (1); (d) background-corrected SERS spectra (excitation wavelength 532 nm) of commercial chitosan.

The Raman spectra of chitosan are characterized by three broad and intense spectral regions located at approximately 800–940, 970–1188, and 1240–1440 cm^–1^. The first region contains characteristic bands near 900 and 912 cm^–1^, which are commonly attributed to NH_2_ wagging vibrations. In the second spectral region, bands observed at approximately 1055, 1118, and 1155 cm^–1^ are associated with C–O, C–O–C, C–C, and C–N vibrational modes originating from the polysaccharide backbone of chitosan. The third broad spectral region includes characteristic peaks near 1280, 1340, and 1380 cm^–1^ related to deformation vibrations of CH, CH_2_, and CH_3_ groups. In particular, the band near 1380 cm^–1^ is commonly associated with CH_3_ vibrations of residual amide groups. In addition, all spectra exhibited a characteristic amide-related band near 1665 cm^–1^ corresponding to C=O–NHR vibrations. Raman features have previously been reported for semicrystalline chitosan materials and were associated with glucosamine-ring vibrations and hydrogen-bonded polysaccharide organization (Table S1, Supporting Information).^4,27–29^

In contrast to conventional Raman measurements, SERS analysis produced spectra with substantially improved signal intensity and spectral resolution, together with effective suppression of the fluorescence background. The fluorescence intensity was considerably higher for chitosan synthesized from Black Soldier Fly pupa shells, reaching approximately 3–5 times greater intensity compared to commercial chitosan (Figure 7c). This behavior is likely associated with residual organic compounds, pigments, or structurally heterogeneous components remaining after the extraction and synthesis process. Variations in chitosan Raman spectra are known to depend strongly on the acetylation degree, molecular organization, and hydrogen-bonding environment of the polymer. Therefore, we analyzed commercial chitosan as a reference for experimentally synthesized chitosan in our work.

In comparison to bulk Raman spectra, the SERS spectrum displays increased spectral resolution and markedly higher intensity for bands associated with the polysaccharide framework.

Prominent features in the 1000–1450 cm^–1^ range are assigned primarily to C–O stretching, C–O–C glycosidic vibrations, C–N stretching, and CH deformation modes of the glucosamine units. Prominent scattering bands around 480–530 cm^–1^ correspond to endocyclic pyranose ring deformations. A sharp peak near 895–900 cm^–1^ is assigned to the equatorial C–H deformation of the β-glycosidic linkage. Strong bands in the 1040–1090 cm^–1^ region reflect skeletal C–O–C stretching and C–N single-bond vibrations, confirming the retention of the main saccharide backbone postsynthesis. The strong bands observed near ∼1150 cm^–1^ and in the 1375–1450 cm^–1^ region correspond to glycosidic bridge vibrations and bending modes of CH_2_ and CH_3_ groups, respectively, suggesting that segments of the polymer backbone are near the metal surface. A notable enhancement at 1380 cm^–1^ is attributed to symmetric CH_3_ and C–N modes (Amide III). The band near 1590–1610 cm^–1^ corresponds to primary amine −NH_2_ scissoring and bending modes. Additional bands in the 1200–1300 cm^–1^ region and near ∼1590 cm^–1^ are attributable to vibrations involving primary amine groups, indicating their participation in adsorption onto the metallic nanostructure (Figure 7c,d and Table 3). Residual Carbonyl Modes (near 1650–1670 cm^–1^) with a weak Amide I (C=O stretching) intensity confirm a high degree of deacetylation across both samples.

**Table 3:** Comparison of Characteristic Vibrational Features in Conventional Raman and SERS Spectra of Chitosan

wavenumber (cm^–1^)	Raman of bulk chitosan	SERS spectrum	interpretation
650–800	weak ring deformation bands	weak–moderate peaks	backbone modes are weakly enhanced unless adsorbed
∼890 to 900	β-glycosidic linkage band (C–H deformation)	present but not dominant	polymer chain signature retained, linkage state
1000–1100	broad C–O stretching band	multiple sharp peaks	selective enhancement of ring and C–O modes
∼1150	strong C–O–C glycosidic vibration	enhanced, often split	indicates backbone near the surface
1200–1300	amide III/C–N modes	strong enhancement	NH_2_ group proximity or interaction of amino-containing groups with the Ag surface
∼1320 to 1340	CH bending	enhanced	orientation-dependent enhancement
∼1375 to 1390	CH_3_ deformation (acetyl)	strong peak	residual acetyl groups close to the surface (amide III)
1420–1450	CH_2_ bending	very strong peaks	typical dominant SERS region
∼1590	NH_2_ bending/primary amine	may shift or broaden	possible interaction with the Ag surface
>1500	weak in Raman	variable intensity	surface interactions/chemical effects

Variations in relative band intensities and minor frequency shifts compared with normal Raman spectra can be attributed to surface-selection effects inherent to SERS, whereby vibrations associated with functional groups directly interacting with the substrate are preferentially enhanced.^30^ Consequently, the observed spectrum reflects the structure of surface-adsorbed chitosan rather than that of the bulk material. These results demonstrate that SERS provides detailed molecular information for chitosan, despite its strong intrinsic fluorescence.

Several differences between normal Raman and SERS spectra were observed, including changes in relative peak intensities, peak broadening, and minor spectral shifts. These effects are characteristic of surface-enhanced Raman scattering and are attributed to surface-selection mechanisms, where vibrational modes associated with functional groups directly interacting with metallic nanoparticles become selectively amplified. The reproducibility of SERS measurements was evaluated by acquiring spectra from different regions of the chitosan samples, and the obtained results for the difference estimation are presented in Figure S2 (Supporting Information). Although the overall spectral profiles remained similar, variations in peak intensity and slight shifts in peak position were observed between different measurement points. These differences may be associated with local variations in chitosan–AgNP interactions and with variations in the local electromagnetic enhancement across the SERS-active surface.^31^ Local SERS variability may also be influenced by the structural heterogeneity of chitosan, including differences in acetylation, hydrogen bonding, and polymer-chain organization.^32^

The observed spectral regions are generally consistent with previous Raman characterization of chitosan, in which bands in the approximately 800–900, 1030–1150, and 1320–1450 cm^–1^ regions were associated with glucosamine-ring, glycosidic-backbone, C–N, and CH-related vibrations.^33^

The comparative analysis of SERS spectra revealed substantial differences between commercial chitosan and chitosan synthesized from Black Soldier Fly pupa shells (Figure 8 and Table 4). Commercial chitosan exhibited a more complex spectral profile with a larger number of narrow and well-resolved bands, whereas synthesized chitosan demonstrated fewer but more intense and broader spectral features. These differences suggest variations in the supramolecular organization, molecular packing, and adsorption behavior at the metallic surface.

**Figure 8:**
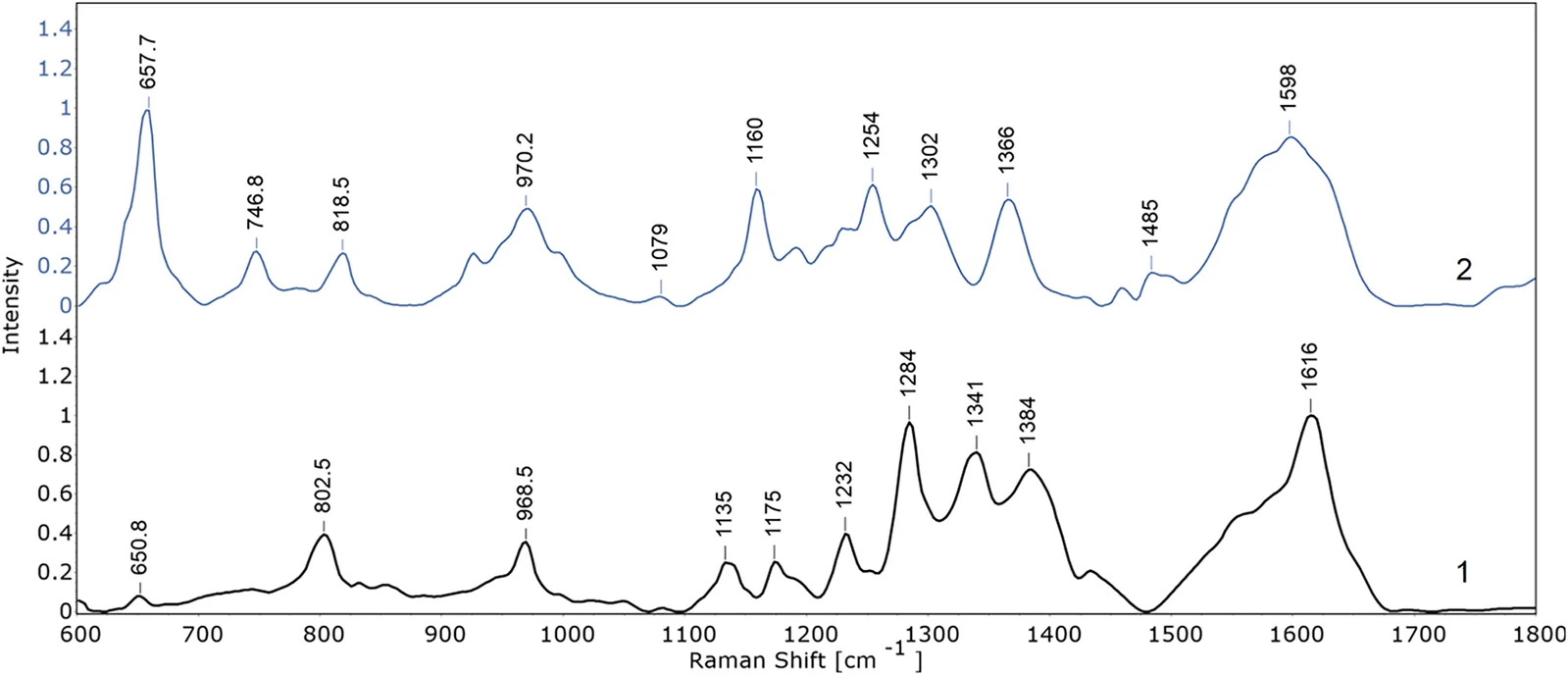
Mean background-corrected SERS spectra of chitosan synthesized from Black Soldier Fly pupa shells (1) and commercial chitosan (2), calculated from spectra acquired at 25 different positions on the SERS substrate for each sample.

**Table 4:** Prominent SERS Peak Positions, Relative Intensities, and Vibrational Assignments of Commercial and Synthesized Chitosan

peak (cm^–1^)	commercial intensity	synthesized intensity	vibrational assignment
∼658	1.000 (dominant)	0.053 (very weak)	pyranose ring deformation
∼804 to 807	0.133–0.162	0.397 (strong)	ring/C–H rocking
∼967 to 969	0.492	0.355	glycosidic ring skeleton
∼1159	0.599	0.072	C–O–C glycosidic stretch
∼1255	0.617	0.203	C–N stretch/amide III
∼1285	0.421	0.970 (dominant)	CH_2_ twisting/amide III
∼1337 to 1339	0.106	0.813	C–H bending/C–N stretch
∼1364 to 1367	0.540	0.584	CH_3_ deformation/amide III
∼1599 to 1615	0.855 (dominant)	1.000	NH_2_ deformation/possible interaction with Ag surface/amide II

Several characteristic Raman bands also exhibited noticeable spectral shifts between the two samples.^33^ For example, the bands near ∼818 and ∼1598 cm^–1^ in commercial chitosan shifted toward ∼803 and 1616 cm^–1^ in synthesized chitosan, respectively. Additionally, several peaks present in commercial chitosan were absent or significantly broadened in the synthesized material. Such spectral behavior may reflect differences in hydrogen-bonding networks, acetylation degree, chain conformation, and local orientation of glucosamine units relative to the plasmonic surface.

### Key Structural and Conformational Differences

Comparison of the SERS spectra revealed pronounced differences in the relative intensities and positions of several characteristic vibrational bands of commercial and synthesized chitosan (Table 4). The primary amine deformation region was prominent in both samples, with bands observed at approximately 1598 cm^–1^ for commercial chitosan and 1616 cm^–1^ for the synthesized material. The relatively strong contribution of amino-group-associated vibrations in the SERS spectra suggests that −NH_2_ groups may represent important interaction sites between chitosan and the AgNP surface. However, the simultaneous enhancement of vibrations associated with the polysaccharide backbone indicates that the interaction is unlikely to involve the amino groups exclusively and is more consistent with multiple contact points between the polymer chain and the AgNP surface.

The pronounced SERS response of amine-associated vibrations, including bands in the approximately 1380 and 1600 cm^–1^ regions, further suggests that amino groups contribute to the interaction of chitosan with the AgNP surface. Their accessibility may facilitate interactions between the polymer and the metal surface, although the observed SERS intensities cannot be interpreted as a direct quantitative measure of the surface-coordination affinity.

Marked differences were also observed in vibrations associated with the saccharide backbone. The commercial chitosan exhibited its highest relative SERS intensity at approximately 658 cm^–1^ (*I* = 1.000), assigned to pyranose-ring deformation, whereas the corresponding band was considerably weaker in the synthesized sample (I = 0.053). Similarly, the C–O–C glycosidic-linkage-associated band at approximately 1159 cm^–1^ was more prominent in the commercial sample (*I* = 0.599) than in the synthesized chitosan (*I* = 0.072). In contrast, the synthesized material exhibited strong relative scattering at approximately 1285 cm^–1^ (*I* = 0.970) and 1339 cm^–1^ (*I* = 0.813), associated mainly with CH_2_ twisting and C–H deformation vibrations, whereas the corresponding bands were less intense in the commercial sample (*I* = 0.421 and 0.106, respectively). These differences indicate distinct local vibrational environments of the polysaccharide chains in the two materials and may reflect differences in molecular organization, hydrogen-bonding interactions, residual acetyl groups, and interactions with the SERS-active surface.

The shift of the prominent amino/amide-associated band from approximately 1598 cm^–1^ in commercial chitosan to 1616 cm^–1^ in the synthesized sample, together with changes in its relative intensity, further indicates differences in the local environment of amino-containing groups. These SERS observations are consistent with the presence of accessible primary amino groups in the synthesized chitosan; however, the band position and intensity alone cannot be used to quantitatively determine the degree of deacetylation or the protonation state.

To provide a quantitative estimate of the SERS signal enhancement obtained for chitosan, we calculated an intensity-based relative enhancement ratio (ER). The conventional SERS enhancement factor (EF) is generally defined by comparing the SERS and normal Raman intensities while accounting for the corresponding number of molecules contributing to the measured signals. In the present study, however, reliable determination of the number of chitosan molecules probed under the two measurement conditions was not feasible because of the heterogeneous particulate nature of the samples and their interaction with the nanostructured SERS surface. Therefore, a conventional EF was not calculated for Chitosan.

Instead, an experimental relative enhancement ratio was estimated from the CH_2_/CH_3_ deformation region (1375–1450 cm^–1^), which was detectable in both conventional Raman and SERS spectra. The measured signal intensities were approximately (*I*_SERS_ = 35,600) counts and (*I*_Raman_ = 400) counts, giving

$$\mathrm{ER}=I_{\mathrm{SERS}}/I_{\mathrm{Raman}}=35,600/400=89$$

Thus, under the respective experimental acquisition conditions, the SERS measurement produced an approximately 89-fold higher signal intensity in this spectral region than conventional Raman spectroscopy. This value should be regarded as an experimental intensity ratio rather than a conventional molecularly normalized SERS enhancement factor, particularly because the Raman and SERS spectra were acquired using different laser powers and acquisition times.

For comparison, the performance of related Ag-based SERS substrates was quantitatively demonstrated in our previous studies. A related silver nanostructured substrate enabled detection of analytes at very low concentrations, including the picomolar range,^34^ while an AgNP-based SERS substrate investigated in another study exhibited an experimentally determined enhancement factor of approximately 10^5^.^35^ These previously reported results provide quantitative context for the performance of the Ag-based SERS approach but should not be interpreted as EF or detection limit values determined for chitosan in the present study.

## Conclusions

Chitosan synthesized from black soldier fly (*H. illucens*) pupa shells was successfully characterized and compared with commercial chitosan using SEM, XRD, FTIR, Raman, and surface-enhanced Raman scattering (SERS) spectroscopy. The synthesized chitosan exhibited a more porous and heterogeneous morphology and showed distinct structural and spectroscopic characteristics compared to the commercial material.

Conventional Raman measurements were strongly affected by fluorescence background, which limited the resolution of weaker vibrational features. SERS measurements enabled the acquisition of well-resolved spectra containing characteristic bands associated with glycosidic linkages, polysaccharide backbone vibrations, amino groups, and residual acetyl functionalities. Notably, SERS enabled the acquisition of well-resolved chitosan spectra using substantially lower laser power (0.1 vs 30 mW) and a shorter acquisition time (5 vs 90 s) than conventional Raman spectroscopy, although the different acquisition conditions preclude a direct quantitative comparison of signal intensities.

Comparative SERS analysis revealed clear spectral differences between commercial and synthesized chitosan in the band intensities, widths, and positions. These spectral variations indicate differences in the local molecular environments of the two materials and may be influenced by factors such as hydrogen bonding, molecular organization, residual acetyl groups, and interactions of chitosan with the AgNP surface. Together with the SEM, XRD, and FTIR results, the SERS data provide complementary information about the structural and spectroscopic differences between the commercial and synthesized chitosan samples.

Overall, this study demonstrates the applicability of SERS as a complementary spectroscopic approach for the characterization of chitosan and provides spectral information for further studies of structurally heterogeneous chitosan materials.

## Supplementary Material



## Notes

Photograph of the chitosan dispersion after sedimentation (Figure S1); representative commercial chitosan SERS spectra acquired at two substrate positions (Figure S2); and assignments of characteristic Raman bands of chitosan (Table S1) (PDF)
