# Supplementary material for: Surface-Enhanced Raman Spectroscopic Characterization of Chitosan Extracted from Black Soldier Fly Pupa Shells
Source: ACS Omega. 2026 Oct 26;11(44):68880–92. doi: 10.1021/acsomega.6c08530 (PMC13756726; doi:10.1021/acsomega.6c08530)
Supplement: Supplementary file 1 [file ao6c08530_si_001.pdf]

Supporting Information

Surface-Enhanced Raman Spectroscopic Characterization of Chitosan Extracted from Black Soldier Fly Pupa Shells

Nora Slekiene[1,6*], Zineb Chiki[2], Ingrida Bruzaite[3], Juozas Padgurskas[4], Valentinas Snitka[5]

[1] Pharmacy and Pharmacology Centre, Institute of Biomedical Sciences, Faculty of Medicine, Vilnius University, M.K. Čiurlionio str. 21/27, Vilnius LT-03101, Lithuania;

[2] Signals, Systems and Components Laboratory (SSCL), Faculty of Sciences and Techniques, Sidi Mohamed Ben Abdellah University, Route Imouzzer, BP 2202, Fez 30000, Morocco; zineb.chiki@usmba.ac.ma

[3] Department of Chemistry and Bioengineering, Faculty of Fundamental Sciences, Vilnius Gediminas Technical University, Sauletekio Av. 11, LT-10223 Vilnius, Lithuania; ingrida.bruzaite@vilniustech.lt

[4] Faculty of Engineering, Vytautas Magnus University (VMU), Studentų str. 15, LT-53362, Akademija, Kaunas, Lithuania; juozas.padgurskas@vdu.lt

[5] Research Centre for Microsystems and Nanotechnology, Kaunas University of Technology, Studentu str. 65, Kaunas LT-51369, Lithuania; vsnitka@ktu.lt

[6] Translational Health Research Institute, Faculty of Medicine, Vilnius University, Žaliųjų Ežerų str. 2, Vilnius LT-08018, Lithuania;

* Correspondence: nora.slekiene@mf.vu.lt; Tel.: +37068506281

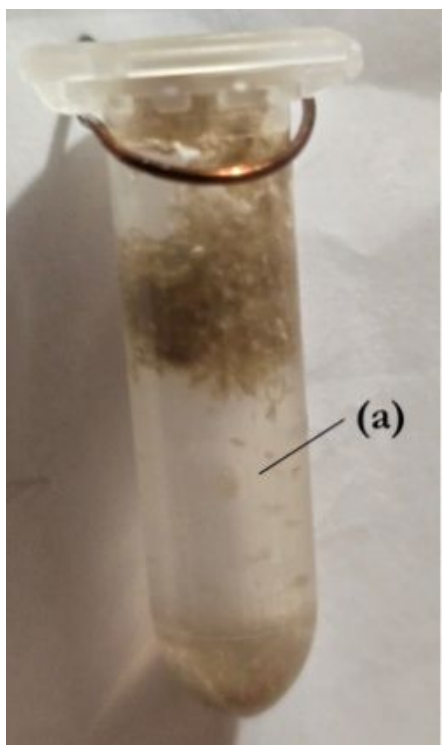

Figure S1. Photograph of the chitosan dispersion after 10 min of sedimentation, showing separation of larger sedimented particles from the suspension used for SERS sample preparation.

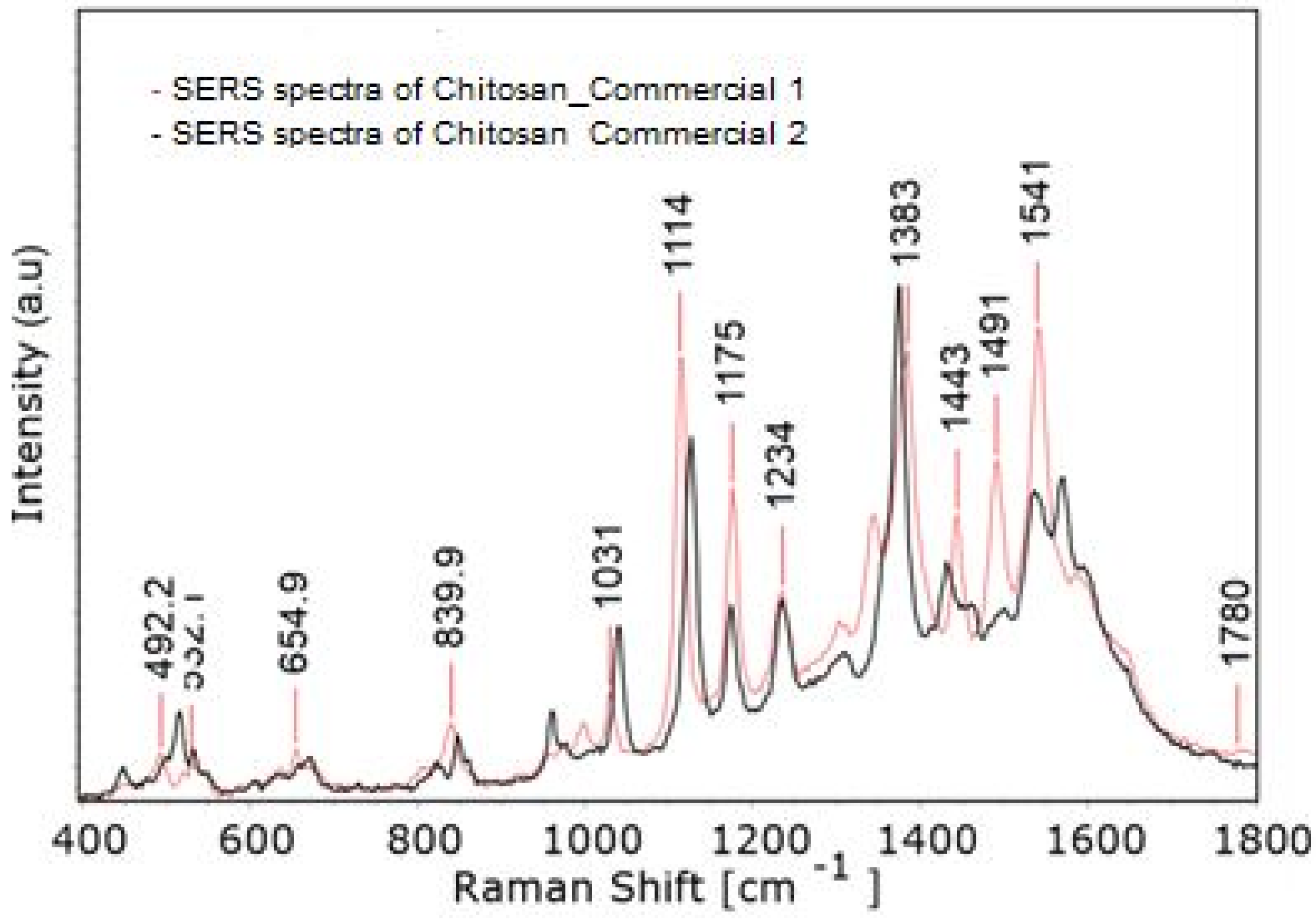

Figure. S2. Representative SERS spectra of commercial chitosan acquired at two different positions on the same SERS substrate: position 1 (red) and position 2 (black).

Table S1. Assignment of characteristic Raman bands of chitosan

Wavenumber (cm^{-1})	Assignment	Structural origin	Interpretation/significance
~650–700	Skeletal C–C–O bending	Polymer backbone	Low-frequency deformation of polysaccharide framework
~750–780	Ring deformation modes	Pyranose ring	Skeletal vibration of the glucosamine sugar ring
~890–900	β-(1→4) glycosidic linkage	C–O–C bridge between monomers	Characteristic band of chitosan chain connectivity
~1000–1010	Ring breathing vibration	Pyranose ring	Indicates intact cyclic sugar structure
~1030–1050	C–O stretching	Secondary alcohol groups (C–OH)	Backbone vibration sensitive to hydrogen bonding
~1080–1105	C–O stretching / C–O–C vibration	Polysaccharide backbone	Common band in carbohydrate polymers
~1145–1160	Asymmetric C–O–C stretching	Glycosidic bridge	Confirms polymer linkage integrity
~1328	C–N stretching + N–H bending (Amide III)	Residual acetamide groups	Indicates remaining acetyl functionality
~1320–1340	CH bending + Amide III contributions	Glucosamine unit	Sensitive to molecular conformation
~1375–1390	CH_3 symmetric deformation	Acetyl methyl group	Marker of residual N-acetyl groups (degree of acetylation)
~1415–1450	CH_2 bending (scissoring)	Backbone methylene groups	Typical vibration of polysaccharide chains
~1550–1600	NH_2 bending / Amide II	Primary amine groups	Characteristic of deacetylated chitosan
